# Usability Evaluation of a VibroTactile Feedback System in Stroke Subjects

**DOI:** 10.3389/fbioe.2016.00098

**Published:** 2017-01-24

**Authors:** Jeremia P. Held, Bart Klaassen, Bert-Jan F. van Beijnum, Andreas R. Luft, Peter H. Veltink

**Affiliations:** ^1^Biomedical Signals and Systems, MIRA – Institute for Biomedical Technology and Technical Medicine, University of Twente, Enschede, Netherlands; ^2^Division of Vascular Neurology and Neurorehabilitation, Department of Neurology, University Hospital of Zurich, Zurich, Switzerland; ^3^Cereneo, Center for Neurology and Rehabilitation, Vitznau, Switzerland; ^4^Centre for Telematics and Information Technology, University of Twente, Enschede, Netherlands

**Keywords:** stroke, rehabilitation, inertial sensing, daily life, technology assessment, vibrotactile, arm usage

## Abstract

**Background:**

To increase the functional capabilities of stroke subjects during activities of daily living, patients receive rehabilitative training to recover adequate motor control. With the goal to motivate self-training by use of the arm in daily life tasks, a sensor system (Arm Usage Coach, AUC) was developed that provides VibroTactile (VT) feedback if the patient does not move the affected arm above a certain threshold level. The objective of this study is to investigate the usability of this system in stroke subjects.

**Method:**

The study was designed as a usability and user acceptance study of feedback modalities. Stroke subjects with mild to moderate arm impairments were enrolled. The subjects wore two AUC devices one on each wrist. VT feedback was given by the device on the affected arm. A semi-structured interview was performed before and after a measurement session with the AUC. In addition, the System Usability Scale (SUS) questionnaire was given.

**Results:**

Ten ischemic chronic stroke patients (39 ± 38 months after stroke) were recruited. Four out of 10 subjects have worn the VT feedback on their dominant, affected arm. In the pre-measurement interview, eight participants indicated a preference for acoustic or visual over VT feedback. In the post evaluation interview, nine of 10 participants preferred VT over visual and acoustic feedback. On average, the AUC gave VT feedback six times during the measurement session. All participants, with the exception of one, used their dominant arm more then the non-dominant. For the SUS, eight participants responded above 80%, one between 70 and 80%, and one participant responded below 50%.

**Discussion:**

More patients accepted and valued VT feedback after the test period, hence VT is a feasible feedback modality. The AUC can be used as a telerehabilitation device to train and maintain upper extremity use in daily life tasks.

## Introduction

To gain independence and increase the quality of life, inpatient neurorehabilitation is usually necessary for hemiparetic stroke subjects (Kollen et al., 2006). The functional capabilities of these patients are assessed using standardized tests, which are intended to predict functional performance after discharge. However, the power of this prediction is poor (Bussmann et al., 2009). Therefore, daily life monitoring of movement quality and quantity would help in guidance of therapy. We previously developed a monitoring solution using a full body inertial sensor suit (Veltink et al., 2014; Klaassen et al., 2015b), with resulting metrics capable of objectifying the quality of movement of stroke subjects. Monitoring in poststroke patients demonstrated that while patients are capable of performing movements during the clinical assessments, they often do not use their affected arm in daily life (van Meulen et al., 2016). These results suggest that capability and arm training does not automatically translate into usage of the affected arm. An unobtrusive coaching system for arm usage during daily life might be able to motivate arm movement in these patients.

In addition to the INTERACTION project, a reduced sensor system was developed with the objective to coach and motivate stroke subjects in remembering to use their affected arm during daily life activities. This Arm Usage Coach (AUC) includes two inertial sensors and one VibroTactile (VT) device. The objective here is to investigate if VT feedback is accepted and the usability of the AUC in stroke subjects during simulated daily life activities. The development of the first prototype and the evaluation with healthy subjects is described in Klaassen et al. (2015a). This paper is a usability study of the first prototype with stroke patients.

## Materials and Methods

### Study Overview

**Figure 1:**
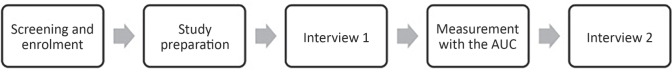
**Flowchart of the study**.

This study was designed as a usability study, conducted at the University Hospital Zurich, to investigate the usability and the acceptance of the AUC. Stroke subjects with mild to moderate arm impairments were enrolled. A semi-structured interview was performed at enrollment, including a questionnaire, to assess the preference of different types of feedback modalities, e.g., VT, visual, and acoustic feedback among stroke subjects. Then, a measurement session was performed using the AUC to let subjects experience VT feedback, responsive to their arm activity and the overall usage of the device. Afterward another semi-structured interview was done, and the System Usability Scale (SUS) (Brook, n.d.) questionnaire was applied to evaluate the system’s usability. An overview is shown in Figure 1.

### Participant Selection

Stroke subjects (above 18 years old) with a unilateral ischemic or hemorrhagic stroke and residual hemiparesis after completion of inpatient rehabilitation were enrolled between March and April 2016. Stroke subjects were required to have a mild to moderate arm impairment with a Fugl-Meyer Assessment upper extremity (FMA-UE, score range 0–66) score higher than 22 (Fugl-Meyer et al., 1975). Additional exclusion criteria were as follows: if the participant has: (1) a major untreated depression, (2) a major cognitive or communication deficits, (3) a major comprehension or memory deficits, (4) major medical comorbidity, (5) severely impaired sensation, (6) sever neglect, and (7) suffering from comprehensive aphasia. Furthermore, the aim for this usability study is to include 10 participants.

### Preparation of the Study

The participants gave written informed consent in accordance with the declaration of Helsinki. The Cantonal ethics in Zurich gave approval in using the VT feedback system (nr. 06-2016). Demographic data of the participant (including age, gender, stroke event, work status, technical background, left or right handed, affected side, and arm dimensions) were documented. Furthermore, vibration sense on the affected arm was assessed using the Revised Nottingham sensory assessment (on the wrist) (Stolk-Hornsveld et al., 2006).

### Preinterview

A semi-structured interview was performed with each participant before the measurement intervention. The questions, with multiple answering options, are listed in Table 1.

**Table 1:** **Questions during pre-interview**.

*#*	Question	Answering options
1	Do you use a self-tracking device?	Yes/No. If yes, what type? Smartphone, wrist band, walking tracker, sleeping mat, other…
2	Do you have any experience with getting feedback?	Yes/No. If yes, by whom? Therapist, doctor, relatives, friends, other…
3	Do you get therapy for the upper extremities?	Yes/No
4	What kind of feedback do you prefer?	Visual, acoustic, vibrotactile, none
5	When should the feedback be applied?	Every 15 min, per hour, every second hour, if the arm is not moving, one time per day, none…
6	Should the information about the feedback be send to the clinician?	Yes/No

### AUC Overview

The AUC is composed of two inertial sensors (Xsens B.V.^1^) (each weights 27 g), an Elitac (Elitac B.V.^2^) VT actuator (weighting 200 g), and a laptop (Klaassen et al., 2015a). Both sensors are wirelessly connected *via* an Xsens dongle, utilizing the Awinda protocol, and the Elitac system *via* Bluetooth. The inertial sensors are worn on each wrist of the participant. The Elitac VT actuator is placed, with Velcro on the affected arm of the participant (Figure 1). The laptop is operating a software program for providing feedback, including analysis of the sensor data, a decision feature, and feedback.

A mandatory starting pose is required from the participant, which is used as a reference pose to compute arm activity by using a metric called the difference acceleration vector (DAV) (Klaassen et al., 2015a). The length of the DAV *d*(*t*) is calculated by subtracting a reference gravitational acceleration vector *g*(*t*), obtained from the sensor data captured during the reference pose, from the current acceleration vector *a*(*t*) of the sensor during daily life movements, and taking the norm of the resulting vector.

$$d\left(t\right)=\sqrt{{\left(a_{x}\left(t\right)-g_{x,t0}\right)}^{2}+{\left(a_{y}\left(t\right)-g_{y,t0}\right)}^{2}+{\left(a_{z}\left(t\right)-g_{z,t0}\right)}^{2}}$$

Finally, the mean DAV is calculated over a 1-s time period of measurement data. The final decision of determining whether a certain mean DAV can be seen as arm movement activity is based on more complex algorithms, as explained in Klaassen et al. (2015a). These decision-making algorithms can be personalized by the following two input parameters of the software, namely, (1) threshold of arm activities (between 0 and 9) and (2) the ratio between the affected and non-affected arm usage (0–1, where 1 means the affected side should be used in the same amount as the non-affected side). The outcome parameters of the algorithms are amount of arm usage (when exceeding the threshold mentioned above, for the left and right arm as percentage of combined arm usage) and the amount of feedback provided over time. A default set of input parameters is used for the software for each participant (threshold = 8 m/s^2^ and ratio = 1). The VT feedback is given at 158.3 ± 2.4 Hz and is given for only 489 ms (300 ms duration, and 189 spin-up and down time of the vibration motor to reach the vibration intensity).

### Measurement Protocol

At the start of the measurement, participants were asked to don the wristbands, which include inertial sensor holders, then click the sensors into the holder and finally mount the VT actuator on the Velcro-wristband on the affected side. Then, the participants were instructed to stand in a comfortable neutral position. This will be the reference position in which arm activity is detected (Klaassen et al., 2015a).

Next, a selection of tasks, listed in Table 2, is performed by the participant twice in a specific measurement area. This measurement area consists of one room (18 m^2^) including a table and a chair, with a door leading to a 15 m long hallway. This set of tasks is performed twice, one time where the VT feedback device is OFF and a second time where the device is turned ON for later comparison.

**Table 2:** **Activity protocol**.

#	Tasks
1	Sit in a chair behind a table in the ARAT test room
2	Stand up and walk to the door
3	Open the door, walk through it to the hallway and close it again
4	Walk 15 m in the hallway
5	Turn around
6	Walk 15 m
7	Open the door, walk through it to the ARAT test room and close it again
8	Walk to the table
9	Move objects from A to B according to the ARAT assessment test in standing
10	Take a seat in a chair

**Figure 2:**
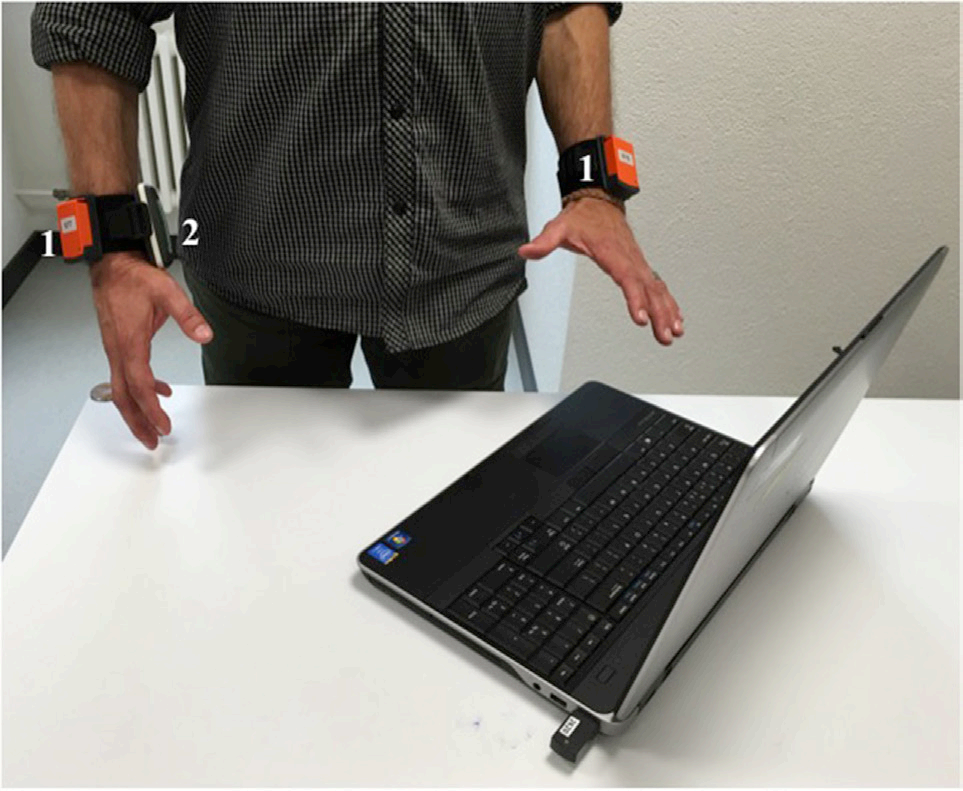
**Right impaired stroke subject with Arm Usage Coach prototype**. (1) Inertial sensors; (2) Elitac VibroTactile actuator.

Tasks 1, 2, 3, 7, 8, and 10 are based on the protocol presented by van Meulen et al. (2015). The tasks were specifically designed for measuring stroke subjects performing simulated activities of daily living. Participants were instructed to stand up from the chair, walk to a door, open the door, walk through the door, and close the door. Then, the participants were instructed to walk in the hallway for 15 m, turn around and walk 15 m back to the door, open, walk through the door, close the door, and walk to a table. On this table (height 75 cm), four blocks (10, 2.5, 5, and 7.5 cm^3^), a cricket ball, a sharpening stone, a drinking glass, and a marble were placed. Participants were asked to grasp each object and place them on a shelf. This combined set is part of the ARAT (Lyle, 1981) assessment while standing. After all, items were placed on the shelf, and the participants were instructed to sit down in a chair. After the measurement, arm usage and the amount of feedback that is given were presented in a visual graph on a computer screen, as shown in Figure 2.

### Postinterview

A semi-structured interview was done after the measurements. Two questionnaires were presented to the participants: (1) a custom-made questionnaire as listed in Table 3 and (2) the SUS (Brook, n.d.). The SUS is a well-established 10-item scale, designed to evaluate usability (effectiveness, efficiency, and satisfaction) of technical devices. Questions were scored on a five-point Likert-type scale ranging from “strongly agree” to “strongly disagree.” Combined scores were translated to a range of 0–100, with a higher score meaning better usability (Brook, n.d.). SUS scores above 90 s reflect best imaginable usability, 85 excellent, 71 good, and 50 suggest fair usability. Scores below 50 indicate that using the product or intervention in practice with will be limited due to low compliance (Bangor et al., 2008, 2009). An additional customized questionnaire (Table 4) was designed to gain more insight into the patient’s preferences in terms of feedback after using the AUC and if they would like to use the AUC at home to increase arm function in daily life.

**Table 3:** **Custom questionnaire during the post interview**.

*#*	Question	Answering options
1	What kind of feedback would you prefer?	Visual, acoustic, vibrotactile, none
2	When should the feedback be applied?	Every 15 min, per hour, every second hour, if the arm is not moving, one time per day, none…
3	Should the information about the feedback be send to the clinician?	Yes/No
4	Would you use a device like the Arm Usage Coach (AUC)?	Yes/No
5	When would you use the AUC?	Daily, Weekly
6	Do you think the AUC could compliment your standard therapy?	Yes/No

**Table 4:** **Participant’s characteristic**.

*P*	Gender^a^	Impaired side	Dominant side	Age	Month post stroke	mRS	FMA-UE	FMA-UE (proximal)	FMA-UE (distal)	Vibration sense^b^
1	M	Left	Right	54	6	1	57	31	26	1
2	M	Left	Right	69	35	2	46	24	22	1
3	F	Left	Right	57	31	3	54	29	25	2
4	M	Right	Right	59	142	3	46	30	16	0
5	M	Left	Right	75	39	1	61	32	27	2
6	M	Right	Right	22	15	1	65	36	29	1
7	M	Left	Left	50	20	2	64	35	29	1
8	F	Right	Right	45	42	3	34	26	8	1
9	F	Right	Left	48	33	1	40	28	12	1
10	M	Left	Right	38	26	2	56	30	24	1
Mean				52	39	1.9	52.3	30.1	21.8	
Std				± 15	±38	± 0.9	±15.1	± 3.7	±7.3	

*^a^Male/female. ^b^Vibration Sense Wrist (0: absent; 1: impaired; 2: normal)*.

*mRS, modified Rankin Scale (0–6 points); FMA-UE, Fugl–Meyer Assessment upper extremity (0–66 points| proximal 36 points | distal 30 points)*.

## Results

### Participant Enrollment

Ten subjects of an ischemic stroke (39 ± 38 months after the event) were recruited in the University Hospital Zurich. Four out of 10 participants wore the AUC on the dominant, impaired arm. Six participants had arm FMA-UE score of larger or equal to 48 points; four participants showed poor to limited arm function (FMA-UE ≤ 47 points). Details of each participant are listed in Table 4. Eight participants had impairments in vibration sense (>64 Hz) on the wrist, at the radial and ulnar styloid process and between the processes. Six participants reported to have a technical occupational background. Seven participants have used self-tracking devices before, for example, a pulse watch and walking trackers. One participant used an activity tracker worn on the wrist to monitor his arm movements during daily life.

### Preinterview Results

The results from the questionnaire given during the preinterview are listed in Table 5. Seven participants had experience with self-tracking devices, e.g., wrist band, walking trackers, or chest strap to measure heart rate. Seven participants mentioned that they have experience with feedback on arm movement provided by relatives, friends, therapists, or self-tracking devices. Eight participants preferred acoustic or visual over VT feedback based on their experience. Four participants mentioned that they would like to receive feedback hourly or when the arm is not moving, one participant every 15 min, and one patient once daily. All participants agreed on sharing the feedback information with a clinician.

**Table 5:** **Results interview 1**.

#	Question	Results
1	Do you use a self-tracking device?	Yes: 7; No: 3
2	Do you have any experience with getting feedback?	Yes: 7; No: 3
3	Do you get therapy for the upper extremities?	Yes: 5; No: 5
4	What kind of feedback would you prefer?	Visual: 2; acoustic: 6; vibrotactile: 3; none: 0
5	When should the feedback be applied?	Every 15 min: 1; per hour: 4; every second hour: 0; if the arm is not moving: 4; one time per day: 1; none: 0
6	Should the information about the feedback be send to the clinician?	Yes: 10; No: 0

### Measurement Results with the AUC

**Figure 3:**
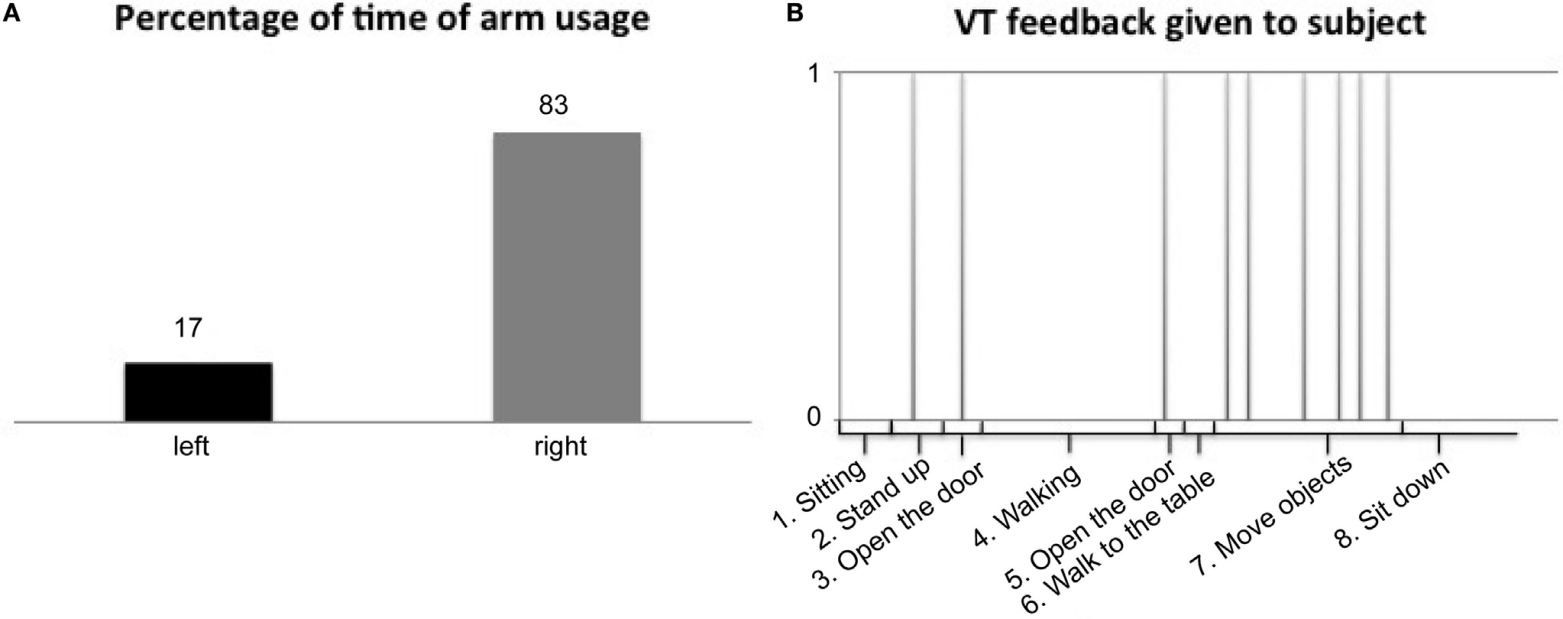
**Examples of arm usage and VibroTactile (VT) feedback results**. **(A)** Percentage of time of arm usage and **(B)** VT feedback over time.

All stroke subjects had hand/wrist function (Page et al., 2015) (FMA-UE distal > 8 points, out of 30 points) and were able to done and doff the wristbands, attach the sensors to sensor holders, and mount VT actuator on the wristband, without any additional devices. In Table 6, a summary of the measurement results are listed (over all participants), including arm usage (in percentage of time of combined left/right arm usage) for the impaired and non-impaired arm and the amount of VT feedback. In addition, an example of arm usage and VT feedback as shown by the AUC is shown in Figure 3. Each participant was able to perform the measurement session (length 15 min) and got VT feedback from the device (on average 6 ± 2 times). Overall, the non-impaired side was used in 57 ± 23% of the time during the session compared to the impaired side with 43 ± 24% of the time. Participants did not report to have any obstruction of the device during their activities. One participant did not feel the VT feedback during the simulated daily life activities, which was congruent with the perception impairment of the participant (Table 1).

**Table 6:** **Summary of the measurement results**.

	Impaired arm usage (%)	Non-impaired arm usage (%)	Difference impaired/non-impaired	Amount of feedback
Average	43	57	−15	6
SD	24	23	47	2

### Postinterview

#### Custom Questionnaire

The results from the questionnaire given during the postinterview are listed in Table 7. After the measurement session, nine out of 10 participants mentioned that they like VT actuation as a feedback modality. More so, seven participants liked and found the VT feedback intuitive when the affected arm was not moving. In total, nine participants would utilize the AUC on a daily basis. All participants would share data generated by the system with a clinician. All participants indicated that they would use the AUC as an addition to their routine therapy in everyday life. Furthermore, participants liked the unobtrusiveness of the VT feedback and that the surrounding environment cannot recognize the feedback. Nine out of 10 participants found the VT feedback helpful, when they do not move the impaired arm.

**Table 7:** **Results custom questionnaire during interview 2**.

#	Question	Results
1	What kind of feedback would you prefer?	Visual: 3, acoustic: 0, vibrotactile: 9, none: 0
2	When should the feedback be applied?	Every 15 min: 0; per hour: 3; every second hour: 0; if the arm is not moving: 7; one time per day: 1; none: 0
3	Should the information about the feedback be send to the clinician?	Yes: 10; No: 0
4	Would you use a device like the Arm Usage Coach (AUC)?	Yes: 10; No: 0
5	When would you use the AUC?	Daily: 9; Weekly: 1
6	Do you think the AUC could compliment your standard therapy?	Yes: 10; No: 0

#### SUS Results

**Figure 4:**
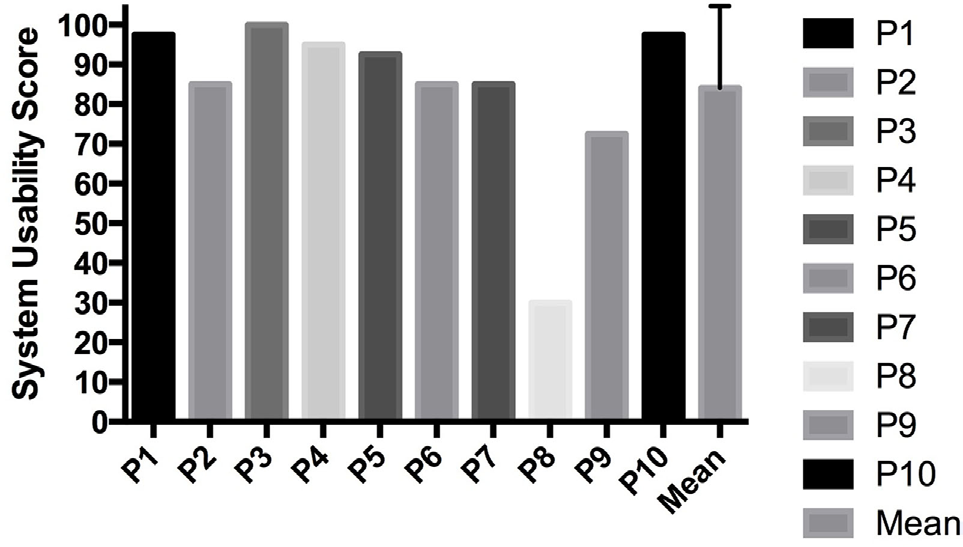
**System Usability Scale results**.

On average, patients reported a SUS score of 84 (± 20.7) out of 100 points indicating excellent usability (Figure 4) (Bangor et al., 2009). Eight participants scored above 80, one between 70 and 80, and one participant reported poor usability below 50. This individual had the worst FMA-UE score (≤ 40). Nine participants reported in the SUS that they would use the system frequently.

## Discussion

The objective of this study was to investigate if VT feedback is accepted and usability of the AUC in stroke subjects during a daily life activities simulation. Based on the inclusion criteria, all included stroke subjects were able to move there affected hand and were able to mount the AUC to the wrist. In total, 70% of the participants said that they would like to obtain feedback when the impaired arm was not moving during certain activities. This indicates good acceptance of the device. In total, 9 out of 10 participants were able to feel the vibration on their impaired arm. Therefore, it appears that participants accepted VT feedback. All participants reported that they would like to use the device, that it complements their current therapy, and that they prefer to share the data with a clinician.

Patients agree to send the data from the AUC to a care professional to check on their progress and address this during therapy sessions, this could help to adapt the rehabilitation for upper extremity to the patients needs. This indicates that AUC could be used as a telemonitoring and -rehabilitation devices for upper extremity. The usability reported with the SUS was high, with 84 out of 100 points on average for all 10 participants. The usability of the AUC is therefore classified as excellent according to Bangor et al. (2009). The AUC however could, according to patients, be improved by being smaller and waterproof. It is increasingly unlikely that new usability problems will be uncovered by including more stroke subjects (Virzi, 1992).

Physical activity coaches, who mostly use accelerometers, implement different feedback strategies (in form of graphs, push-messages, VT feedback) to encourage active behavior during daily life (Cabrita et al., 2015; Achterkamp et al., 2016). In stroke, feedback is used to investigate certain interventions (e.g., constrained induced movement therapy) (Bonifer et al., 2005; Harris et al., 2009; Shi et al., 2011), to correct postures of patients during specific tasks (Ding et al., 2013), or to improve motor learning capabilities (Lieberman and Breazeal, 2007). Moreover, many studies showed that multimodal feedback strategies, proved to be effective to improve performance of patients in various tasks and scenarios (Burke et al., 2006; Prewett et al., 2006; Causo et al., 2012; Trejo-Gabriel-Galan et al., 2016). Most of these studies are performed in a lab environment, and therefore have a reduced interest in the social context of the patients. We designed the AUC based on two feedback strategies: first, knowledge of performance, implemented by VT feedback, which is given during simulated daily life tasks in this study. The second is knowledge of results, which is given through visual feedback where the arm usage is shown in a bar graph, in percentage of the left and right arm and the number of feedbacks within a given time period. This combines the real-time VT feedback with post-visual feedback. This differs by most work done in multimodal strategies, which in most studies gives a combined (near) real-time feedback. In our design, we aimed for an unobtrusive and wearable design during daily life, without the direct need of smartphone apps to make it more applicable and intuitive for stroke patients. Acoustic feedback is obtrusive in social settings and was not implemented. Furthermore, our visual feedback needs longer data processes in order to “make sense” (arm usage); therefore, there is no need for direct visual feedback. Systems to train the upper extremity function with VT feedback in stroke patients have been previously developed (Kapur et al., 2009; Acuna et al., 2010; Bark et al., 2011; Hung et al., 2015). It is known that intensive training after stroke has a positive effect in clinical outcome, but the effect of VT feedback on arm function is unclear (Hung et al., 2015). The AUC could provide the opportunity to increase the arm usage in daily life, thereby training intensity and time by providing VT feedback.

We did not observe an effect of the AUC on arm usage of the impaired side. This is due to the short observation period. Because the main objective here was to test the usability and acceptance of the system, hence a short measurement time was selected.

The combination of monitoring and training stroke patients in daily life with VT feedback is new and could be realized by using a smaller, waterproofed version of the AUC. Computational tasks should be performed on the sensor, rather than on a laptop or a smartphone.

Based on the results of this usability study, an efficacy study, with extended protocol and pre defined outcome parameter, could evaluate the impact of VT feedback on the stroke subjects arm movements in daily life activities.

## Author Contributions

BK and JH drafted the manuscript and analyzed the data. JH performed patient measurements inside the hospital with remote supervision of BK. BK prepared, tested, and optimized the measurement system. B-JB helped in drafting the manuscript and assisted with data interpretation. AL, PV, and B-JB supervised the research. All the authors read, corrected/commented, and approved the final manuscript.

## Conflict of Interest Statement

AL is a scientific advisor for Hocoma AG (Volketswil, Switzerland), which develops rehabilitation technology. The remaining authors declare that the research was conducted in the absence of any commercial or financial relationships that could be construed as a potential conflict of interest.

## Footnotes

^1^http://xsens.com.

^2^http://Elitac.nl.
